# Human IgG responses to *Anopheles gambiae* immunogenic salivary proteins in urban and rural populations of Burkina Faso: biomarkers of exposure to malaria vector bites

**DOI:** 10.1186/s13071-025-06792-1

**Published:** 2025-05-19

**Authors:** Michaël Kaboré, Yéri Esther Hien, Dado Jean Noël Koussé, Fatimata Thiombiano, Mireille Ouédraogo, Abdoul Rahamani Nikiema, Enock Ibrango, Nicolas Ouédraogo, Mariama K. Cherif, Sylvain Ilboudo, Toussaint Rouamba, Guillaume Sylvestre Sanou

**Affiliations:** 1 Laboratoire de Biochimie et Immunologie Appliquées (LaBIA), École Doctorale Sciences et Technologies, Université Joseph KI-ZERBO, 03 BP 7021 03 Ouagadougou, Burkina Faso; 2 Centre National de Recherche et de Formation sur le Paludisme (CNRFP), Ouagadougou, Burkina Faso; 3 Institut de Recherche en Sciences de la Santé (IRSS), Ouagadougou, Burkina Faso; 4 Université Nazi BONI, Bobo-Dioulasso, Burkina Faso; 5 Unité de Recherche Clinique de Nanoro (URCN), Nanoro, Burkina Faso; 6 Laboratoire de Développement du Médicament, Centre d’Excellence Africain de Formation, de Recherche et d’Expertises en Sciences du Médicament, Université Joseph KI-ZERBO (LADME/CEA-CFOREM/UJKZ), Ouagadougou, Burkina Faso

**Keywords:** Malaria, IgG antibodies, Salivary proteins, *An. gambiae* bites, Burkina Faso

## Abstract

**Background:**

Malaria control would be greatly facilitated by the development of new tools for rapidly assessing malaria transmission intensity. In malaria-endemic areas such as Burkina Faso, human populations are frequently exposed to immunomodulatory salivary components injected during mosquito blood feeding. Numerous studies have examined parasite immunity; however, there are few data available on vector immunity as a means of assessing malaria transmission in sub-Saharan Africa. The present study aims to compare IgG-specific response to salivary gland extracts (SGE) of *Anopheles gambiae* (*An. gambiae*) in populations living in urban and rural areas in Burkina Faso.

**Methods:**

A cross-sectional descriptive study was carried out in two sites, Ouagadougou city and Sapouy village, where blood samples (*n* = 676) from children (0–15 years) and adults were collected. After *An. gambiae* salivary protein isolation, the antibody (IgG) response to those SGE was evaluated by enzyme-linked immunosorbent assay (ELISA), representing a proxy of *Anopheles* exposure. The difference in antibody concentrations between groups was tested using parametric tests (Student’s *t*-test and analysis of variance [ANOVA]) and the nonparametric Mann–Whitney *U* (Wilcoxon rank-sum) test. All differences were considered significant at *P* < 0.05.

**Results:**

The study population consisted of 63.0% males and 37.0% females (average age = 31.2 ± 17.8 years). IgG antibodies against *An. gambiae* salivary protein were detected in all study participants. Urban participants demonstrated a greater mean IgG level to *An. gambiae* bites than rural (*P* < 0.0001). The mean IgG level was higher in secondary school children compared with primary school children (*P* < 0.0001). Organic cotton farmers held higher IgG to *An. gambiae* bites than conventional cotton farmers (*P* = 0.0027).

**Conclusions:**

The evaluation of IgG specific to mosquito salivary gland extracts as immunological biomarkers in populations in Burkina Faso allowed us to show that the human anti-SGE IgG level to *An. gambiae* bites is strongly influenced by the living environment and the use of insecticides in agriculture.

**Graphical Abstract:**

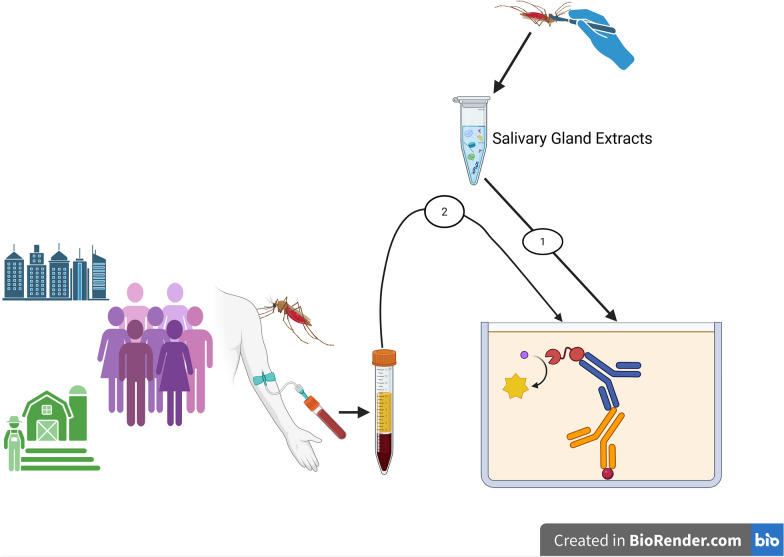

## Background

Mosquitoes are involved in numerous human diseases, such as malaria, that kill or debilitate millions of people each year [1]. Despite the progress achieved in controlling malaria, it remains a major public health problem, contributing to morbidity and mortality, especially in children under 5 years of age in sub-Saharan Africa (80%). According to the latest World Malaria Report, there were 249 million cases of malaria in 2022 against 244 million in 2021 [2]. The estimated number of deaths attributable to malaria was 608,000 in 2022 against 610,000 in 2021. About 94% of malaria cases (233 million) and 95% of deaths (580,000) from the disease were recorded in the World Health Organization (WHO) African Region [2]. In 2022, Burkina Faso recorded 11,656,675 cases of malaria, including 539,488 cases of the severe form. Unfortunately, 4243 deaths were recorded, of which 2925 were children under 5 years of age [3].

Globally, nearly 2.2 billion people are at risk of malaria [4]. In the context of prevention, several strategies have been implemented, including vector control and vaccines. Vector control is an essential component of malaria control and elimination strategies, as it is highly effective in preventing infection and reducing transmission.

The two primary interventions are long-lasting insecticide-treated nets (LLINs) and indoor residual spraying (IRS), which reduce contact with infected mosquitoes [5, 6]. There are approximately 422 species of *Anopheles* worldwide, but only about 68 act as vectors of *Plasmodium* spp. In sub-Saharan Africa, malaria vectors are classified into three main categories, consisting of about 12 species. *An. gambiae* and *An. funestus* are major vectors of malaria, with a wide geographical distribution, high vectorial capacity, and sporozoite indices greater than 1% [7–10].

Malaria is endemic to Burkina Faso, and populations are frequently exposed to bites from only one or a few *Anopheles* mosquito species, which are the predominant malaria vectors. During the rainy season, *An. gambiae* is the major malaria vector in Burkina Faso [11–14]. Significant differences were observed between urban areas and villages (parasite index: 16% versus indices ranging from 51% to 88%) [15].

Entomological surveillance for malaria is inherently resource-intensive and produces crude population-level measures of vector exposure. Currently, the gold standard measurement of malaria transmission intensity is the entomological inoculation rate (EIR), a population measure defined as the number of infective *Anopheles* mosquito bites a person receives per unit of time [16].

In some individuals, the bite of a blood-sucking arthropod is often followed by a hypersensitivity reaction at the bite site. This is due to the production of IgG and IgE specific to immunogenic salivary proteins [17–20]. Over the past decade, several studies showed that quantifying the antibody (Ab) response to vector saliva in human populations could serve as a pertinent biomarker tool to assess human exposure to vector bites and, consequently, the risk of transmission of vector-borne diseases [21]. In addition, new immuno-epidemiological tools have been developed to assess exposure to mosquito bites at both the population and individual levels [22, 23]. These innovative tools are based on measuring human antibody responses to the salivary proteins of arthropod vectors injected during the bite [23–32].

Studies are being conducted at multiple sites to generate standardized surveillance data that improve understanding of malaria transmission [30, 33, 34] and to monitor and evaluate the impact of interventions to inform malaria control and elimination programs [35]. The IgG response to *An. gambiae* salivary gland extracts (SGE) has been identified and validated as a relevant biomarker of mosquito bites [23, 24, 36]. It is a reliable tool for assessing the spatial and temporal heterogeneity of exposure at both the population and individual levels [10, 37].

Despite the importance of antibodies against *An. gambiae* salivary peptides as a relevant biomarker of mosquito bites [23, 24, 34, 37–40], data on immuno-epidemiological biomarkers of human exposure to *An. gambiae* bites remain relatively scarce in Burkina Faso. These data are needed to better understand and reduce malaria transmission.

For this reason, the present study aims to evaluate and compare the IgG specific response to SGE of *An. gambiae* in populations living in urban areas (children) and rural areas (adults). The IgG specific response to SGE was also assessed across children based on age groups, and in cotton farmers following their agricultural practices (i.e., the use of synthetic pesticides or not).

## Methods

### Study area

**Fig. 1:**
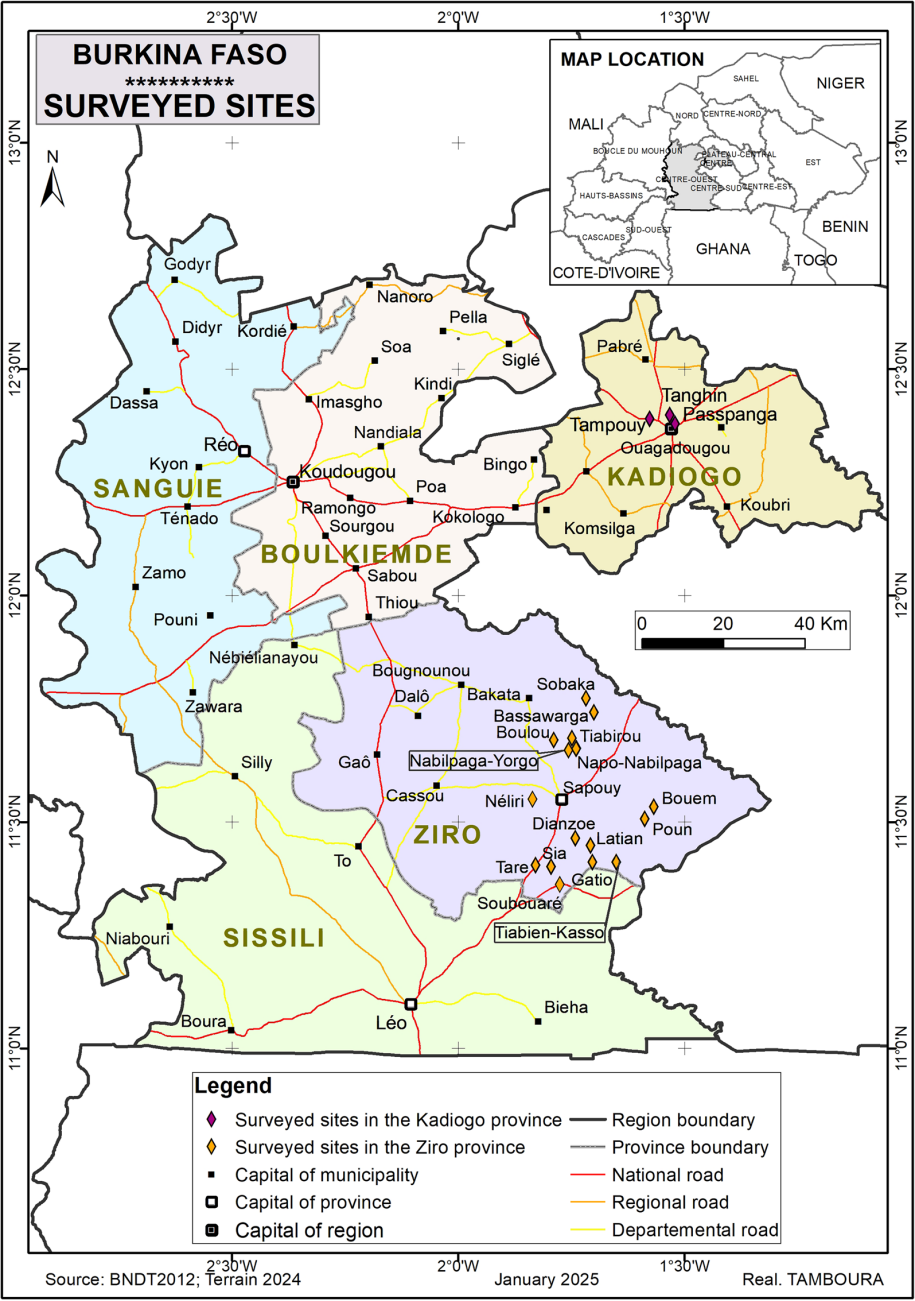
Map highlighting study areas with dots

The study was conducted at two sites: (i) 16 villages located in the province of Ziro (11°33′16″N, 1°46′25″W), 100 km from Ouagadougou; and (ii) Ouagadougou, the capital city of Burkina Faso (Fig. 1). A cross-sectional descriptive study was carried out from September 2020 to March 2021 for the collection of samples from children, and from June to July 2022 for the collection of samples from adult cotton farmers. Conventional cotton farmers used only chemical compounds (synthetic pesticides) such as carbamates, organophosphates, and pyrethroids, while organic cotton farmers used pesticides derived from natural substances (plant extract mixtures, bacteria, and others) to control pests.

### Study design and sample collection

The studies were conducted according to the principles of the Declaration of Helsinki [41] and in full conformity with relevant country regulations. Ethical assessments were obtained in Burkina Faso by: *Comité d’Éthique pour la Recherche en Santé du Burkina Faso (No. 2020-10-217)* and *Institutional Ethics Committee for Health Research of the Research Institute for Health Sciences in Burkina Faso (No. 009–2022/CEIRES of 20 January 2022)*. The study was approved by the Burkina Faso Ministry of Health and Public Hygiene. Site leaders provided prior permission to survey each site.

Adult farmers of organic and conventional cotton were included in Sapouy, and children under 15 years of age attending primary and secondary school in Ouagadougou were included in the study. Participants were selected using a simple random sampling technique, ensuring equal probability for all eligible individuals. Permission was granted for consent, assent, and collection sheets. Validated consent was obtained from a parent or guardian of each participating child, as well as from the adult participants. In addition to parental consent, children aged 12 and over provided their free, informed, and written assent.

During the administration of consent, we provided information about the study, the amount of blood to be collected, the risks of participating in the study, the confidentiality of participants’ information, and the freedom to withdraw consent at any time without consequences.

**Fig. 2:**
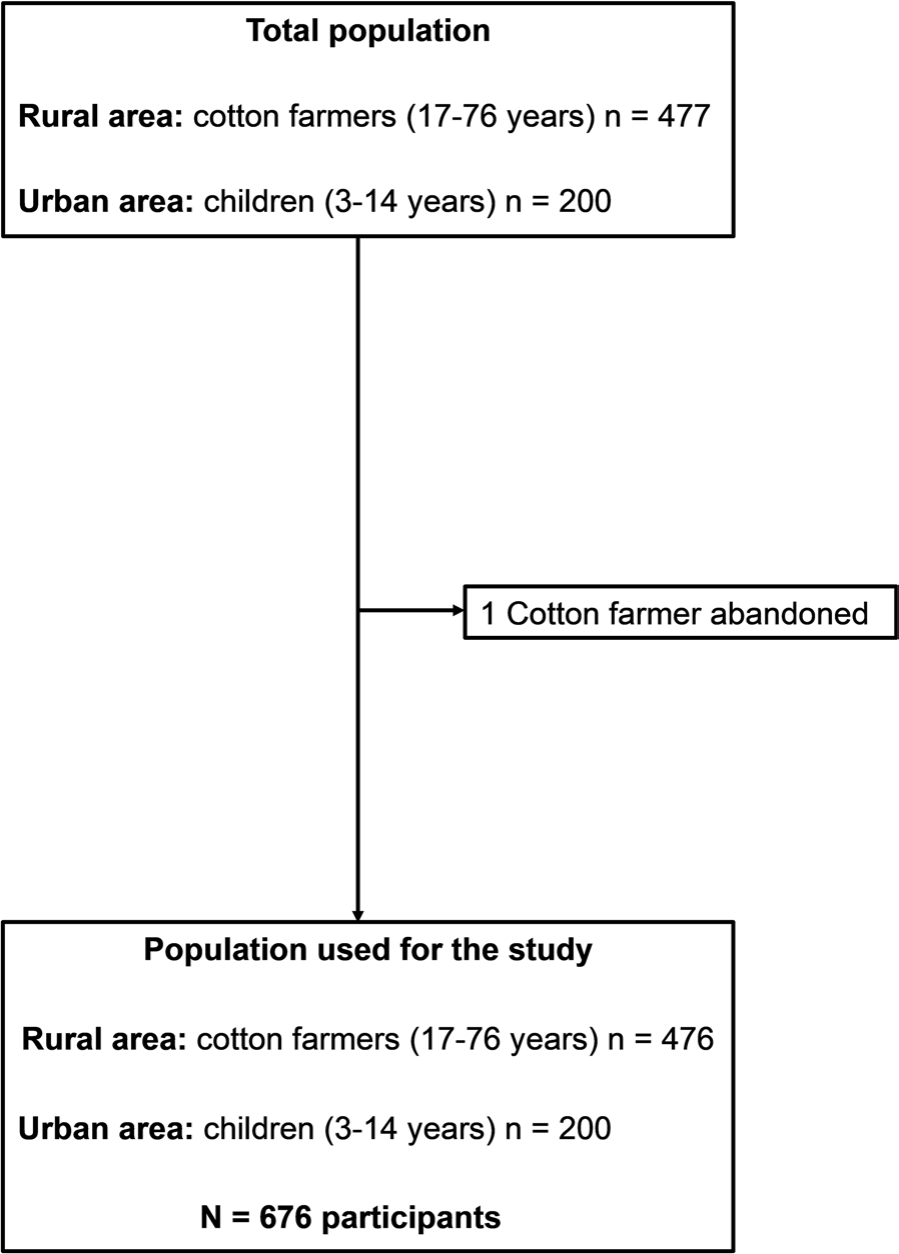
Flowchart of the study population

Overall, blood samples were collected from 200 children aged 3–14 years in urban areas and 477 cotton farmers aged 17–76 years in rural areas. One cotton farmer withdrew from the study. Therefore, 200 children and 476 cotton farmers were included in the analysis (Fig. 2).

Blood samples (5 mL) were collected in tubes containing ethylenediaminetetraacetic acid (EDTA) as an anticoagulant for immunological assays. Thick and thin blood films were prepared for microscopic examination, and plasma samples (aliquots of 150 µL) were stored at −20 °C until analysis.

### Parasitological diagnosis

Thick and thin blood films were air-dried, fixed with methanol, stained with 3% Giemsa, and examined double-blind by two certified microscopists. Discordant readings were re-examined by a third qualified independent microscopist. One hundred high-power fields (HPFs) were examined, and the number of malaria parasites of each species and stage was recorded. The number of parasites per microliter of blood was calculated by assuming 20 white blood cells per high-power field and a fixed white blood cell count of 8000/µL. A blood smear was considered negative if no parasites were observed.

### Salivary gland dissection

The protein extracts were produced from reared *An. gambiae* specimens. Female mosquitoes were anesthetized on ice for 10 minutes before being dissected to remove the salivary glands. Salivary glands were dissected in phosphate-buffered saline [10 mM NaH₂PO₄, 145 mM NaCl (pH 7.2)] and carefully transferred to a microcentrifuge tube in a small volume of phosphate-buffered saline (50 µL). The samples were kept at −80 °C until use.

### Extraction by sonication

Sonication is the process of converting an electrical signal into a physical vibration for various purposes. It is usually performed to disrupt cell membranes and release cell contents for later evaluation. The salivary glands of *An. gambiae* were placed in a sonicator for 10 min at a maximum of 60 Hz. The crude extracts were centrifuged at 15,000 rpm for 15 min. After centrifugation, the proteins were placed into Eppendorf tubes for quantification.

### Protein quantification

Salivary gland proteins were quantified using a NanoDrop spectrophotometer (ND-1000; No. 414). A 1 µL sample of salivary proteins was deposited at the center of the spectrophotometer, and the measurement was performed at 280 nm. The spectrophotometer was connected to a monitor that displayed the amount of salivary protein per microliter (µL).

### Assessment of antibody responses

The enzyme-linked immunosorbent assay (ELISA) was performed following a standardized methodology described in the AIA standard operating procedures, as detailed elsewhere [37]. Venous blood samples were collected in tubes, and plasma was obtained after centrifugation at 3000 rpm for 10 min. Plasma samples were fractionated into aliquots and then frozen at −20 °C until use.

In brief, 96-well Maxisorp micro-assay plates were coated with *An. gambiae* salivary gland extracts (SGE) at 0.1 µg/mL and incubated at 4 °C overnight. The plates were blocked with skimmed milk buffer (3% milk powder in phosphate-buffered saline containing 0.1% Tween 20, abbreviated as 0.1% PBST) for 1 h at room temperature and covered with adhesive paper (Parafilm). This blocking solution was chosen owing to its lower background value. It lines the bottom of the plate, reducing background noise.

After this step, human plasma samples diluted 1/200 (vol:vol), positive and negative controls, and diluted standards of known concentrations were added in duplicate and incubated at room temperature with stirring for 2 h. Then, a specific anti-human IgG (secondary antibody) conjugated to peroxidase (1/100) was added to each well. The plates were incubated with stirring for another hour. The wells were washed three times with PBS between each step.

Bound secondary antibodies for IgG were quantified by adding 100 µL/well of ready-to-use TMB (3,3',5,5'-tetramethylbenzidine) substrate and incubating for 30 min in the dark at room temperature. Following incubation with stop solution (hydrochloric acid), optical density (OD) was read at 450 nm.

Individual results were expressed as the ΔOD value:

ΔOD = ODx − ODn,

where ODx represents the mean individual optical density (OD) value in both wells with *An. gambiae* SGE, and ODn represents the individual OD value for each serum without *An. gambiae* SGE. The ΔOD value of the test sample was converted into arbitrary units using the ADAMSEL program (Microsoft Excel worksheets).

### Statistical analysis

All statistical analyses were performed using STATA™ version 17.0 (Stata Corporation). Parametric tests (Student’s *t*-tests and analysis of variance [ANOVA]) were used for independent groups with normally distributed antibody levels. The nonparametric Mann–Whitney *U* (Wilcoxon rank-sum) test was used to compare IgG levels between two independent groups with non-normally distributed antibody levels. All differences were considered significant at *P* < 0.05.

## Results

Approximately two-thirds (63.0%, 426/676) of the study population were male. The mean age of participants was 31.2 ± 17.8 years. The most represented age group was ≥ 25 years (62.1%, 420/676). More than two-thirds (70.4%, 476/676) of the study population lived in rural areas (Table 1).

**Table 1:** Baseline characteristics of the study participants

Characteristics	Frequency (%)
Gender	
Male	426 (63.0)
Female	250 (37.0)
Sex ratio F/H	0.6
Age (years)	
Mean	31.2 ± 17.8
Range	[3–76]
Median	39.5
Age group	
[0–5]	2 (0.3)
[5–10]	100 (14.8)
[10–15]	98 (14.5)
[15–20]	11 (1.6)
[20–25]	45 (6.7)
≥ 25	420 (62.1)
Profession	
Primary school	141 (20.9)
Secondary school	59 (8.7)
Organic cotton farmers	193 (28.5)
Conventional cotton farmers	283 (41.9)
Area of residence	
Urban	200 (29.6)
Rural	476 (70.4)

### Human IgG response to *Anopheles gambiae* salivary proteins

Human anti-SGE IgG were detected in all samples tested, indicating that all participants were exposed to *An. gambiae* mosquito bites, albeit at different levels of exposure.

Urban participants demonstrated greater exposure to *An. gambiae* bites than rural participants (*P* < 0.0001). Significant differences in mean IgG levels were observed between the two groups of children (primary and secondary school) (*P* < 0.0001). Organic cotton farmers expressed higher IgG levels compared with conventional cotton farmers (*P* = 0.0027). The mean antibody level to antigenic proteins of *An. gambiae* salivary glands was significantly associated with age (*P* = 0.0001). Gender had no significant influence on the mean IgG level against *An. gambiae* salivary gland extracts (*P* = 0.2175) (Table 2).

**Table 2:** Mean level of human IgG response to *Anopheles gambiae* salivary gland extracts

Groups	Frequency	Mean IgG	[95% CI]	Statistical analysis
Primary school	141	0.3	[0.2–0.3]	*z* = 8.411, *P* < 0.0001*
Secondary school	59	1.1	[0.9–1.2]	
Organic cotton farmers	193	0.2	[0.1–0.3]	*t* = 3.0123, *P* = 0.0027*
Conventional cotton farmers	283	0.1	[0.1–0.2]	
Urban	200	0.5	[0.4–0.6]	*z* = −13.811, *P* < 0.0001*
Rural	476	0.2	[0.1–0.2]	
Ages [0–5]	2	0.2	[-0.1–0.6]	*F* = 41.38, *P* = 0.0001*
Ages [5–10]	100	0.3	[0.2–0.4]	
Ages [10–15]	98	0.8	[0.6–0.9]	
Ages [15–20]	11	0.3	[-0.1–0.8]	
Ages [20–25]	45	0.2	[0.1–0.3]	
Ages ≥ 25	420	0.2	[0.1–0.2]	
Male	426	0.3	[0.2–0.3]	*z* = 1.233, *P* = 0.2175
Female	250	0.3	[0.2–0.3]	

*CI* confidence interval

^*^Statistically significant

### Prevalence of *Plasmodium falciparum* among children according to *Anopheles gambiae* exposure

Overall, *Plasmodium falciparum* prevalence detected by microscopy was 4.5% (9/200) in children (Table 3). The mean concentration of the IgG response to *An. gambiae* SGE was higher in *P. falciparum*-positive children (0.6) compared with *P. falciparum*-negative children (0.5). However, the difference was not statistically significant (*P* = 0.7345).

**Table 3:** *Plasmodium falciparum* prevalence according to *Anopheles gambiae* exposure in children

*P. falciparum* prevalence by IgG	Frequency (%)	Mean IgG	[95% CI]	Statistical analysis
*P. falciparum* positive	9 (4.5)	0.6	[0.2–1.0]	*t* = −0.3397, *P* = 0.7345
*P. falciparum* negative	191 (95.5)	0.5	[0.4–0.6]	
*Anopheles gambiae* exposure according to education level in *P. falciparum*-positive children				
Primary school	4 (44.4)	0.2	[0.1–0.3]	*t* = 2.9300, *P* = 0.0220*
Secondary school	5 (55.6)	0.9	[0.3–1.5]	
*P. falciparum* density according to education level in *P. falciparum*-positive children				
		Primary school	Secondary school	
Mean density of *P. falciparum*		975.3 (*n* = 4)	2121.4 (*n* = 5)	*t* = 0.8881, *P* = 0.4040
Range of *P. falciparum*		[268–1743]	[126–5297]	

*CI* confidence interval

^*^Statistically significant

No significant difference in the prevalence or density of *P. falciparum* was observed between children in primary and secondary school (*P* = 0.342 and *P* = 0.4040, respectively) (Table 3).

### IgG responses to *Anopheles gambiae* salivary gland extracts in conventional cotton farmer villages

**Fig. 3:**
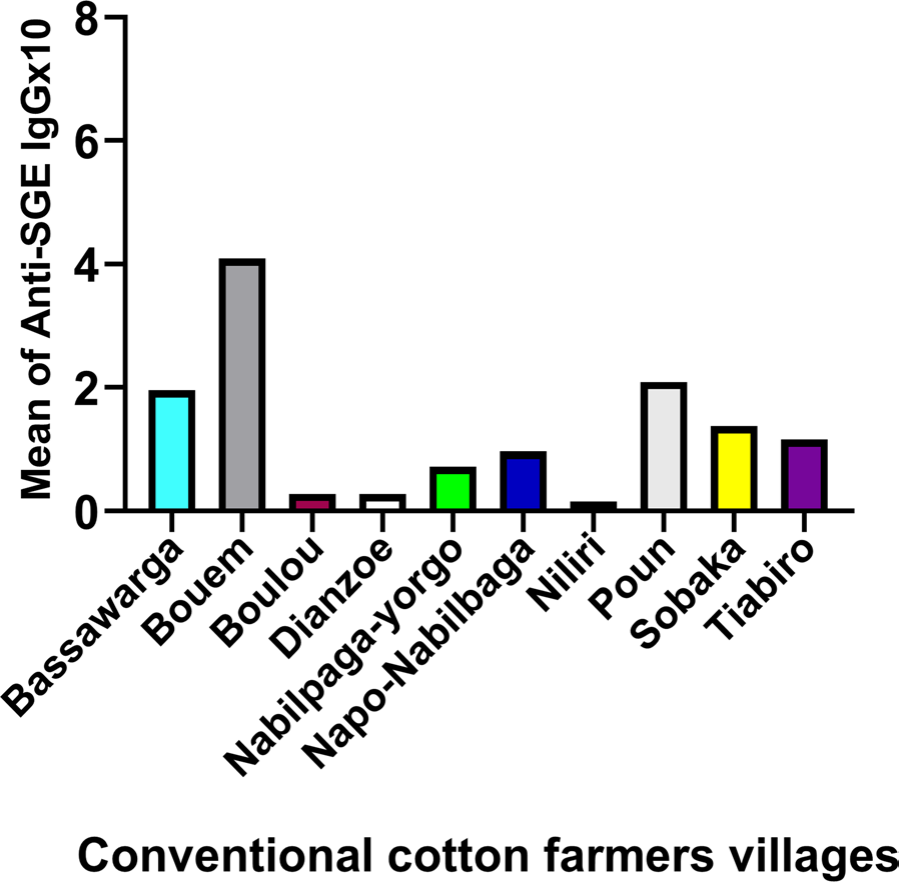
Mean concentration of the IgG response to *Anopheles gambiae* salivary proteins by village. Participants were grouped by village (*n* = 10), and the mean IgG level against *An. gambiae* SGE was calculated using individual IgG levels in each village. In these villages, cotton farmers used only chemical compounds (synthetic pesticides) such as carbamates, organophosphates, pyrethroids, and others to control cotton pests

Among the ten villages where conventional cotton was cultivated, Bouem had the highest mean IgG level, while Niliri had the lowest mean IgG level to *An. gambiae* SGE among conventional cotton farmers (Fig. 3).

### Human IgG responses to *Anopheles gambiae* salivary proteins among organic cotton farmer villages

**Fig. 4:**
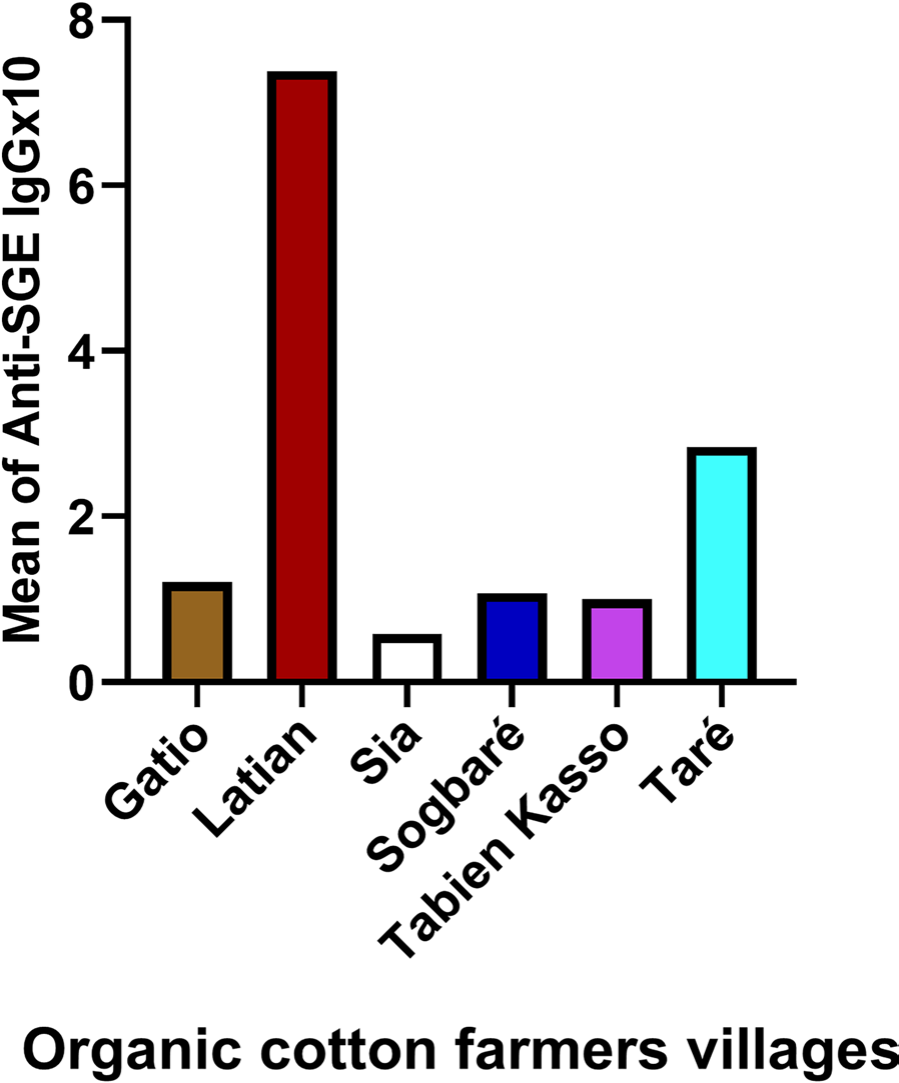
Mean concentration of IgG responses to *Anopheles gambiae* salivary gland extracts by village. Participants were grouped by villages (*n* = 6), and the mean IgG against *An. gambiae* SGE was calculated on the basis of individual IgGs to *An. gambiae* SGE in each village. In these villages, farmers cultivated cotton without using chemical compounds, relying solely on pesticides derived from natural substances (plant extract mixtures, bacteria, and others) to control pests

Among organic cotton farmers, the mean IgG level to *An. gambiae* SGE was highest in Latian village and lowest in Sia village (Fig. 4).

## Discussion

The serological evaluation of the antibody response to mosquito saliva and its association with exposure to malaria vectors has received increasing attention owing to the limitations of current techniques in estimating malaria transmission [42]. *An. gambiae*-specific salivary polypeptides have been shown to be reliable markers of human exposure to Afrotropical malaria vectors, as demonstrated by studies in Burkina Faso, Tanzania, and Uganda [43–45].

In this study, we used an innovative serological marker tool to quantify human–vector contact and estimate the risk of malaria transmission in endemic areas on the basis of living environment (urban or rural), age (primary or secondary school), and type of agriculture practiced (conventional or organic cotton).

Human anti-SGE IgG was detected in all samples tested, indicating that all participants were exposed to *An. gambiae* mosquito bites, albeit at different levels. Urban participants were more exposed to *An. gambiae* bites than rural participants (*P* < 0.0001), as evidenced by higher average anti-SGE IgG levels in urban areas in our study. In cities, household drinking water storage practices and the discharge of sewage into streets could create potential breeding sites for *Anopheles*. In Ouagadougou, a reservoir has been transformed for vegetable farming and other cultivation, increasing the heterogeneity of urban environments in terms of vegetation and standing water (small puddles and breeding sites), each of which can influence mosquito abundance, particularly *An. gambiae*, the major malaria vector in Africa and a key driver of disease transmission [46–48]. In addition, malaria control strategies may be more difficult to plan and coordinate in urban areas, where the unregulated occupation of space could promote the proliferation of breeding sites for malaria vectors [46, 49, 50]. Previous research suggests that lower-income neighborhoods generally have more standing water due to residential abandonment, garbage dumps, and inadequate sewage systems [48]. Many such poor neighborhoods exist in Ouagadougou, where we collected data, the capital of a low-income country. A limitation of this comparison is the absence of adults in our urban study population and the absence of children among participants in rural areas.

Significant differences in mean IgG levels were noted between the two groups of children (primary and secondary school) (*P* < 0.0001). This result reflects differences in the way immune responses develop in individuals who are continuously exposed to *Anopheles* bites. Anti-SGE IgG levels to *An. gambiae* increased with age in all children (*P* < 0.0001) and in *P. falciparum*-positive children (*P* = 0.0220), with a less intense response in primary school children. This pattern is similar to those observed with salivary antigen proteins of *An. gambiae* [27, 51] and various *P. falciparum* antigens in the same epidemiological setting [52]. In addition, secondary school children stay outside later in the evenings, increasing their exposure to *Anopheles* bites [53].

The mean antibody level to antigenic proteins of *An. gambiae* SGE was statistically associated with age (*P* = 0.0001). The average IgG level increased with age up to the 15–20 year age group before decreasing. This decline could be explained by the high number (*n* = 283) of conventional cotton farmers (ages > 15 years) in our study population. The pesticides used in conventional cotton farming may repel mosquitoes, reducing exposure to mosquito bites.

Our findings demonstrated that the antibody response to *An. gambiae* SGE varies according to age, agricultural practices, and area of residence. Organic cotton farmers exhibited higher IgG levels than conventional cotton farmers, a statistically significant difference (*P* = 0.0027). Agricultural practices likely modulate human–vector contact in our study area. Conventional cotton farmers used only chemical compounds (synthetic pesticides) such as carbamates (e.g., propoxur and bendiocarb), organophosphates (e.g., malathion and fenitrothion), organochlorines (e.g., DDT), pyrethroids (e.g., deltamethrin, permethrin, cypermethrin, and lambda-cyhalothrin), and others to control cotton pests. These same classes of pesticides are widely used in mosquito control programs, including the deployment of insecticide-treated mosquito nets (ITNs) and indoor residual spraying (IRS) of insecticides [54]. These practices likely contribute to lower vector exposure in conventional cotton farmers by repelling mosquitoes. In recent years, the use of insecticides to combat mosquito-borne diseases has increased [55].

We found that *An. gambiae* SGE antibody concentration was positively correlated with malaria infection status in children in Ouagadougou, but this correlation was not statistically significant. Dipomin F. Traoré et al. reported a similar finding in their study [56], whereas other studies showed a statistically significant correlation [30]. The lack of statistical significance in our study could be due to the small sample size or a high level of exposure to *Anopheles* bites.

Bouem village had the highest mean IgG level to *An. gambiae* SGE, while Niliri had the lowest in conventional cotton farmers. In organic cotton farmers, the highest mean IgG level to *An. gambiae* SGE was recorded in Latian village, while the lowest was in Sia village. The reason for the higher vector exposure in these villages is unknown, but it may be influenced by factors such as human behavior, agricultural practices, vector control measures, population movement, and/or immunogenicity characteristics.

Using individual salivary components, such as *An. gambiae* SGE, could simplify and standardize experimental systems, providing insights into the complex relationships between vectors, parasites, and vertebrate hosts.

There are a few weaknesses in our study.

- Unavailability of data from children in rural areas and lack of data from adults in urban areas: these absences can be explained by the fact that the data were collected at different time periods and with different initial objectives: (1) comparison of human anti-SGE IgG according to education level (age) in children; and (2) according to the type of agriculture practiced in rural areas,
- Unavailability of information of vector control parameters in the study population. Indeed, this data was not collected.

## Conclusions

To advance progress toward malaria elimination, the World Health Organization has called for innovative tools and improved approaches to enhance vector surveillance, as well as the monitoring and evaluation of interventions [57].

Our study evaluated human–vector contact using a new tool vector immunity as a means of assessing the relationship between the parasite, vectors, and humans. The results showed a significant difference in the mean anti-SGE IgG level between rural and urban populations. Age and type of agriculture also had a significant impact on the mean level of anti-salivary gland extract IgG of *Anopheles gambiae*.

## Data Availability

Data will be made available on reasonable request.

## Abbreviations

ELISA: Enzyme-linked immunosorbent assay
IgG: Immunoglobulin G
OD: Optical density
CI: Confidence interval
